# Chlorin e6-Loaded PEG-PCL Nanoemulsion for Photodynamic Therapy and In Vivo Drug Delivery

**DOI:** 10.3390/ijms20163958

**Published:** 2019-08-14

**Authors:** Changhee Park, Jihye Yoo, Donghyun Lee, Seok-young Jang, Soonmin Kwon, Heebeom Koo

**Affiliations:** 1 Department of Medical Life Sciences, College of Medicine, The Catholic University of Korea, 222 Banpo-daero, Seocho-gu, Seoul 06591, Korea; 2 Department of Biomedicine & Health Sciences, College of Medicine, The Catholic University of Korea, 222 Banpo-daero, Seocho-gu, Seoul 06591, Korea; 3 Catholic Photomedicine Research Institute, College of Medicine, The Catholic University of Korea, 222 Banpo-daero, Seocho-gu, Seoul 06591, Korea

**Keywords:** nanoemulsion, nanoparticle, polycaprolactone, drug delivery, photodynamic therapy, chlorin e6

## Abstract

We fabricated poly (ethylene glycol)-block-polycaprolactone (PEG-b-PCL) nanoemulsion for drug delivery and photodynamic therapy. PEG-b-PCL effectively stabilized the interface between water and soybean oil, and the resulting nanoemulsion was about 220.3 nm in diameter with spherical shape. For photodynamic therapy (PDT), chlorin e6 (Ce6) was loaded into the nanoemulsion as a photosensitizer (PS). These chlorin e6-loaded PEG-PCL nanoemulsions (Ce6-PCL-NEs) showed efficient cellular uptake and, upon laser irradiation, generated singlet oxygen to kill tumor cells. Particularly, Ce6-PCL-NEs showed prolonged blood circulation and about 60% increased tumor accumulation compared to free Ce6 after intravenous injection to 4T1 tumor-bearing mice. These results demonstrate the promising potential of Ce6-PCL-NEs for efficient PDT and in vivo drug delivery to tumor tissue.

## 1. Introduction

Photodynamic therapy (PDT) is a photochemical method that uses a light-activated drug molecule called a photosensitizer (PS) [1]. Upon irradiation with an appropriate wavelength, PS generates reactive oxygen species (ROS) with sufficient cytotoxicity to destroy target disease cells [2]. In particular, PDT has been applied for the therapeutic treatment of various cancers including those of the bladder, esophagus, prostate, head and neck, renal system, and skin [3,4]. For successful PDT, efficient delivery and accumulation of PS in target tissue and cells are essential. To achieve this purpose, nanoparticle (NP) carriers have been developed and demonstrate promising results [5,6]. NPs can transport drugs without aggregation by chemical conjugation or physical loading [7]. Due to their size, they can increase the circulation time of drugs in the body, preventing unintended early secretion [8]. Particularly, NPs pass through the endothelial lining of the blood vessels in angiogenic sites like tumor tissue because the vessel wall is more permeable than that of normal tissues. The traversing NPs accumulate there along with the aid of weakened lymphatic drainage, an effect that has been called enhanced permeability and retention (EPR) [9]. Furthermore, the surface of NPs can be decorated with biological ligands that bind receptors on target cells and increase the specificity of delivered NPs [10,11]. These advantages have made NPs promising carriers of drugs including PSs [12,13].

For efficient drug delivery, different types of NPs have been fabricated with various materials such as polymers, lipids, gold, iron oxide, silica, and carbon [14]. Among them, nanoemulsion is one of the oldest NP fabrications. It is a biphasic dispersion composed of two liquids, oil and water, which are stabilized by a surfactant [15]. Oil-in-water (O/W) emulsion in particular is widely used for dispersion of hydrophobic molecules in aqueous conditions. Surfactant is an amphiphilic material that reduces interfacial tension between oil and water and plays a pivotal role in maintaining the size and stability of nanoemulsion. Until now, researchers have found or developed various kinds of surfactants including lecithin, sodium deoxycholate, cremophor EL, sodium dodecyl sulfate, and sorbitan monolaurate [16]. Block copolymers based on hydrophilic and hydrophobic polymers are also attractive surfactants for nanoemulsion formation. Previously, Nam et al. introduced poly (ethylene glycol)-block-polycaprolactone (PEG-b-PCL) for providing excellent stability during fabrication of O/W nanoemulsions [17]. PCL has a lower melting point compared to other hydrophobic polymers such as polylactic acid (PLA) or poly (lactic-co-glycolic acid) (PLGA). Therefore, PEG-b-PCL is fully miscible with oil at 80 °C and forms a homogeneous oil/polymer mixture. While cooling to room temperature, PEG-b-PCL migrates to the interface between oil and water and effectively stabilizes the nanoemulsion structure. This PEG-b-PCL nanoemulsion with its high stability has high potential for drug delivery.

In this report, we fabricated PEG-b-PCL nanoemulsions by heating and cooling and applied them to deliver chlorin e6 (Ce6), a representative PS for PDT. The size and shape of these Ce6-loaded PEG-PCL nanoemulsions (Ce6-PCL-NEs) were characterized in vitro. In 4T1 mouse breast cancer cells, their cellular uptake, singlet oxygen generation, and photodynamic effect were also analyzed after laser irradiation. Finally, their in vivo biodistributions and tumor accumulations were analyzed in 4T1 tumor-bearing mouse model after intravenous injection.

## 2. Results

### 2.1. Development and Characterization of Ce6-PCL-NEs

**Figure 1:**
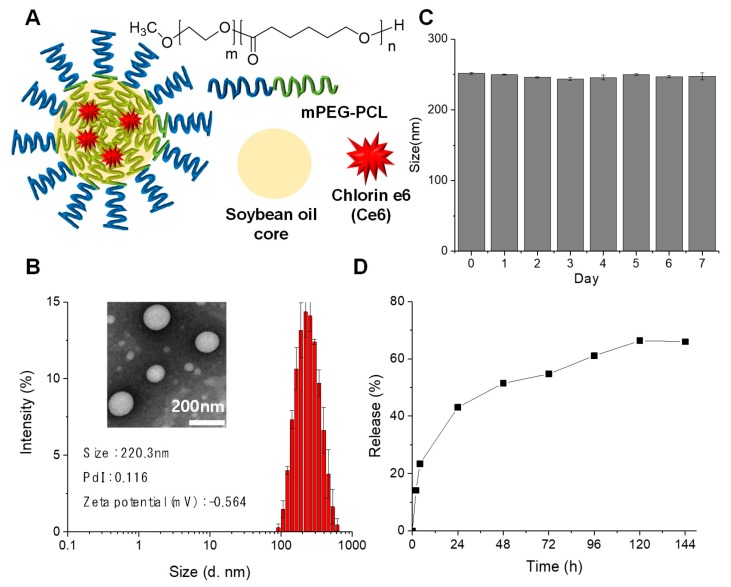
Synthesis and characterization of Ce6-PCL-NE. (**A**) Schematic illustration of Ce6-PCL-NE. (**B**) Size distribution and TEM image of Ce6-PCL-NEs. (**C**) Size change of Ce6-PCL-NEs in PBS (pH 7.4) during one week. (**D**) Release profile of Ce6 from Ce6-PCL-NE for 6 days.

Ce6-PCL-NEs were prepared by a conventional O/W emulsion method using soybean oil as the inner phase. Ce6 was selected as PS due to its high singlet oxygen quantum yield and near-infrared (NIR) wavelength [18]. It was encapsulated into a hydrophobic soybean oil core shielded by amphiphilic PEG-b-PCL (Figure 1A). Ce6 and soybean oil were dissolved in DMSO, and PEG-b-PCL was dissolved in ethyl alcohol. The two solutions were added into water and treated with probe sonicator. Dynamic light scattering (DLS) data showed that the size of Ce6-PCL-NEs was about 220.3 nm, and the zeta potential was measured as –0.564 mV, close to neutral. The morphology of Ce6-PCL-NEs was observed by transmission electron microscopy (TEM), which revealed that they were spherical in shape (Figure 1B). When we measured the size of Ce6-PCL-NEs for one week, we did not observe significant changes showing their good stability (Figure 1C). To analyze drug release of Ce6-PCL-NEs, they were placed into dialysis bags in PBS at physiological pH (pH 7.4) (Figure 1D). After burst release of the Ce6 at the initial time, it was slowly released over five days, showing high stability of the fabricated nanoemulsions.

### 2.2. Cellular Uptake of Ce6-PCL-NE and ROS Generation

**Figure 2:**
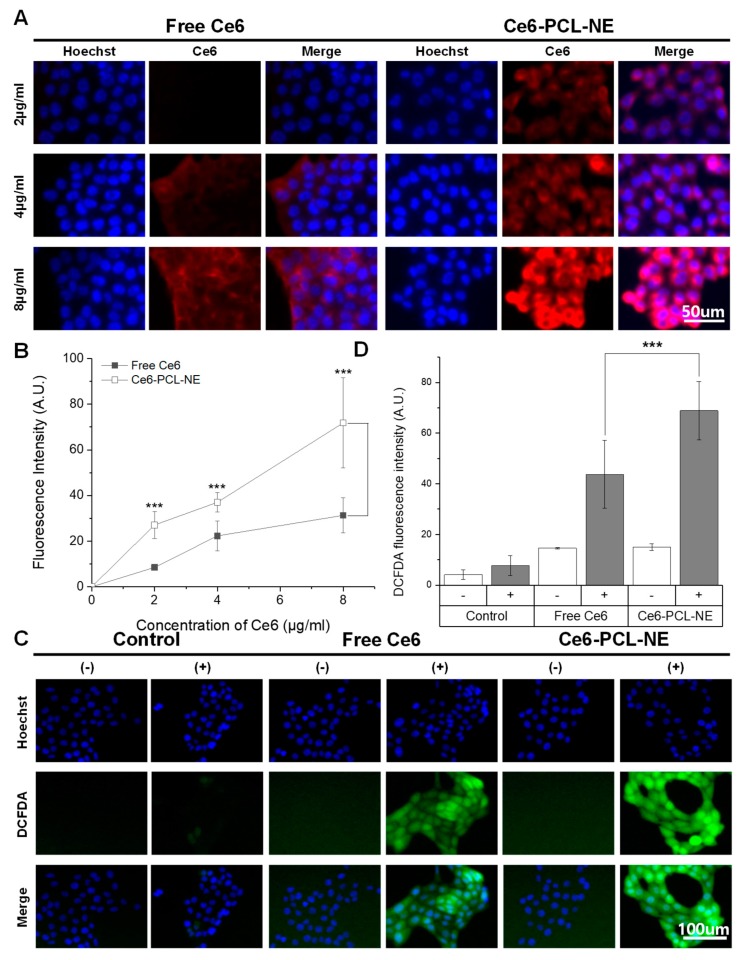
Cellular uptake of Ce6-PCL-NE and ROS generation. (**A**) Fluorescence images of 4T1 tumor cells treated by free Ce6 or Ce6-PCL-NE for 2 h. (**B**) Fluorescence intensity with different concentrations of Ce6. Results represent mean ± s.d. (*n* = 10). *** *p* < 0.001. (**C**) Fluorescence signals of 2’,7’-dichlorofluorescein diacetate (DCFDA) in 4T1 tumor cells treated with free Ce6 or Ce6-PCL-NE for 2 h with or without laser irradiation. (**D**) Fluorescence intensity of DCFDA in (**C**). *** *p* < 0.001. Results represent mean ± s.d. (*n* = 10).

To investigate cellular uptake ability of the Ce6-PCL-NE, 4T1 mouse breast cancer cells were incubated with different concentrations of free Ce6 and Ce6-PCL-NE for 2 h (Figure 2A). The image of Ce6-PCL-NEs in tumor cells showed more intense fluorescence compared to the image of free Ce6 in tumor cells. The fluorescence intensity of Ce6 gradually increased according to concentration in both free Ce6 and Ce6-PCL-NE treatments (Figure 2B). To observe ROS generation from Ce6-PCL-NEs in 4T1 cells upon laser irradiation, we used 2’,7’-dichlorofluorescein diacetate (DCFDA) as ROS probe (Figure 2C). The control group without Ce6 showed no significant ROS generation regardless of laser irradiation. Results were similar in cases of free Ce6 and Ce6-PCL-NEs without laser irradiation. In contrast, both free Ce6 and Ce6-PCL-NE groups showed intense green fluorescence upon laser irradiation, indicating a photodynamic effect and ROS generation. The fluorescence intensity of Ce6-PCL-NE-treated cells was higher than that of free Ce6-treated cells, which had similar cellular uptake data (Figure 2D). These results revealed that, upon laser irradiation, Ce6-PCL-NEs triggered ROS generation in tumor cells for tumor therapy.

### 2.3. Cytotoxicity Test of Ce6-PCL-NE with or without Laser Irradiation

**Figure 3:**
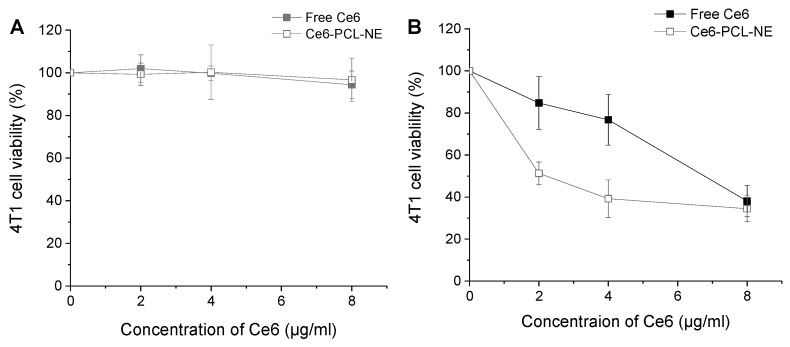
Dark toxicity test of Ce6-PCL-NE and in vitro photodynamic effect. Cell viability based on MTT assay (**A**) in the dark and (**B**) upon laser irradiation in 4T1 cells treated with free Ce6 and Ce6-PCL-NEs for 2 h. Results represent mean ± s.d. (*n* = 6).

The MTT assay was performed to evaluate cell viability after treatment at different concentrations of Ce6-PCL-NEs or free Ce6 in 4T1 cells. In dark conditions, the cytotoxicity of both groups was weak until 8 µg/mL Ce6 concentration (Figure 3A). After irradiation by laser, the cell viability of both groups decreased in a Ce6 concentration-dependent manner. At low concentration, Ce6-PCL-NE showed enhanced tumor cell death by photodynamic effect in comparison with free Ce6 (Figure 3B).

### 2.4. In Vivo Biodistribution of Ce6-PCL-NE in 4T1 Tumor-Bearing Mice

**Figure 4:**
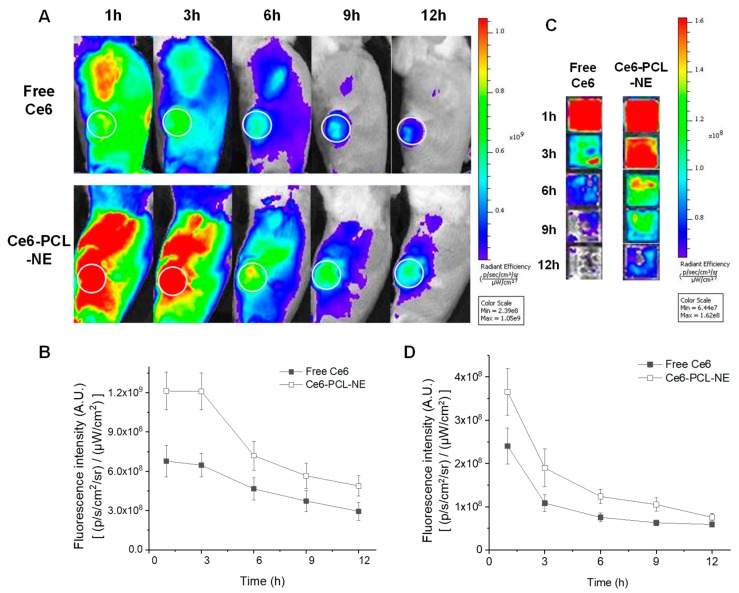
In vivo biodistribution of Ce6-PCL-NE in 4T1 tumor-bearing mice after intravenous injection. (**A**) Whole body NIR fluorescence images of 4T1 tumor-bearing mice after intravenous injection of free Ce6 and Ce6-PCL-NE. (**B**) Quantification of fluorescence intensity analysis at tumor site in (**A**) (*n* = 3). (**C**) NIR fluorescence images of blood from mice in (**A**). (**D**) Quantification of blood fluorescence intensity in (**C**) (*n* = 3).

To observe the biodistribution of Ce6-PCL-NE, free Ce6 and Ce6-PCL-NE were intravenously injected into 4T1 tumor-bearing mice (2.5 mg/kg Ce6 concentration). After injection, real-time whole-body NIR fluorescence images were obtained by an IVIS Lumina XRMS system at different time points. In Ce6-PCL-NE-treated mice, accumulation of Ce6 at the tumor site was relatively higher at all times compared to free Ce6-treated mice (Figure 4A). Twelve hours postinjection, the Ce6 signal in the tumor sites of Ce6-PCL-NE-treated mice was about 1.7-fold higher than in tumor sites of free Ce6-treated mice (Figure 4B). The Ce6 fluorescence in blood from free Ce6 or Ce6-PCL-NE-treated mice was also analyzed by IVIS, indicating that Ce6-PCL-NE has longer blood circulation time than free Ce6 (Figure 4C,D). We expect that prolonged blood circulation and EPR effect might enhance tumor accumulation of Ce6-PCL-NEs.

### 2.5. Ex Vivo Analysis of Resected Organs and Tumor Tissues

**Figure 5:**
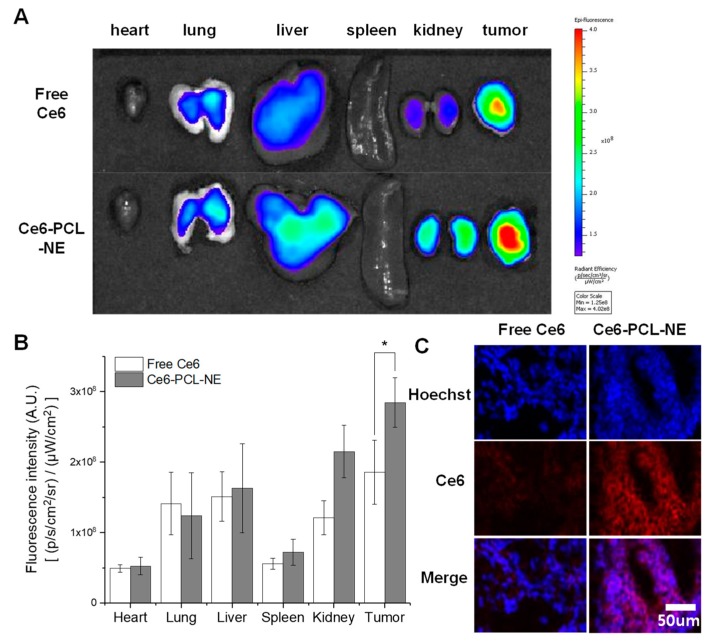
Ex vivo imaging analysis of Ce6-PCL-NE in 4T1 tumor-bearing mice. (**A**) Ex vivo fluorescence images of the dissected tumors and major organs (heart, lung, liver, spleen, and kidney) 12 h post-injection of free Ce6 and Ce6-PCL-NE into 4T1 tumor-bearing mice. (**B**) Quantification of fluorescence intensity of (**A**) (*n* = 3). * *p* < 0.05. (**C**) Fluorescence images of the sliced tumor tissue 12 h after injection of free Ce6 and Ce6-PCL-NE.

At 12 h after intravenous administration of free Ce6 or Ce6-PCL-NE, ex vivo images were obtained from the resected tumor tissues and major organs (heart, lung, liver, spleen, and kidney) (Figure 5A). The fluorescence intensity of Ce6-PCL-NE increased about 60% compared to that of free Ce6 in tumor tissue. Other than tumor, fluorescence intensities were highest in kidney and liver, representative secretory organs (Figure 5B). Frozen sections of the tumor tissues also exhibited stronger fluorescence signal of Ce6-PCL-NE compared to that of free Ce6 (Figure 5C).

### 2.6. Accumulation of Ce6-PCL-NE in Metastatic Tumor Tissues

**Figure 6:**
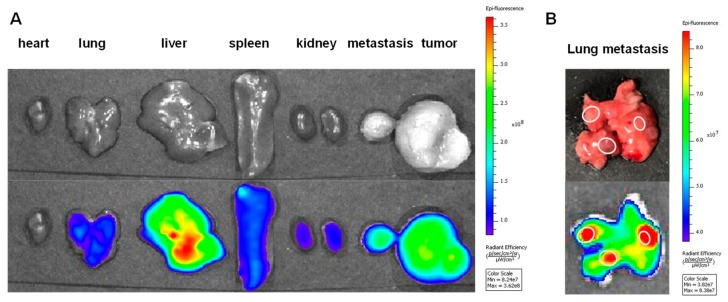
Accumulation of Ce6-PCL-NE in metastatic tumors of 4T1 tumor-bearing mice. (**A**) Ex vivo fluorescence images of the dissected tumors including primary/metastasis tumors and major organs (heart, lung, liver, spleen, and kidney) 12 h post-injection of Ce6-PCL-NE into 4T1 tumor-bearing mice. (**B**) Ex vivo fluorescence image of lung metastasis after intravenous injection of Ce6-PCL-NE into 4T1 tumor-bearing mice.

About two weeks after subcutaneous injection of 4T1 cells into the left flank, unintended metastatic tumors had formed along with the growth of the primary tumor. We injected Ce6-PCL-NE intravenously into one mouse with a secondary tumor (about 150 mm^3^) between the chest and skin. Twelve hours postinjection, ex vivo images of dissected tumor tissues and major organs were acquired and analyzed (Figure 6A). We found that the fluorescence intensity of Ce6-PCL-NEs was much higher in metastatic tumor as well as in the primary tumor compared to normal tissues. Another mouse had lung metastasis and also underwent Ce6-PCL-NE injection. Large tumor nodules were observed on the lung and were colocalized with high fluorescence signals (Figure 6B). These results demonstrated Ce6-PCL-NE as efficiently delivered to not only the primary tumor, but also metastasized tumors.

## 3. Discussion

There have been various studies about tumor-targeting strategies of NPs [19]. Passive targeting based on physical stability and EPR effect and active targeting using biological ligands represent most strategies. In this study, our Ce6-PCL-NE has a size of about 220.3 nm, and its surface charge was near neutral at –0.564 mV. Near neutral surface charge and PEG groups on Ce6-PCL-NEs may reduce aggregation with serum protein. Based on these data, we expected that it would be suitable for prolonged blood circulation and EPR effect-based tumor accumulation [9]. After intravenous injection, Ce6-PCL-NEs showed increased circulation time in blood as shown in the fluorescence images in Figure 4C. Due to the prolonged circulation in blood flow and EPR effect, they also showed increased tumor accumulation compared to free Ce6, as expected (Figure 4A). However, we did not use any biological ligand in the current form of these particles. Therefore, the tumor-targeting ability of the particles will be further enhanced if we modify their surface with suitable ligands such as peptides, antibodies, or aptamers [20]. In case of Ce6-PCL-NE, the concentration of Ce6 was also higher in kidney and liver differently with that of free Ce6. It may originate from the slow excretion of Ce6-PCL-NE due to the longer circulation time in blood as shown in Figure 4C.

Until now, metastasis has been regarded as one of the big hurdles to overcome during tumor therapy. Therefore, drug delivery to metastatic tumors is as important as that to primary tumors. In this study, we used the 4T1 mammary carcinoma cell line, which is highly tumorigenic and invasive [21]. As the primary tumor grew after subcutaneous injection of 4T1 cells into mice, unintended metastasis was found in different organs including lung and liver. Interestingly, we observed high accumulation of Ce6-PCL-NE in these metastatic tumor tissues in lung or other tissue, indicating that Ce6-PCL-NE can deliver drugs to metastatic tumors as well as primary ones, showing promising potential of the particles as drug carriers. We expect that further study with intended lung metastasis model by intravenous injection of 4T1 cells will provide more information [22].

## 4. Materials and Methods

### 4.1. Materials

PEG-b-PCL was purchased from Ruixibio (Xi’an city, China). Chlorin e6 (Ce6) was purchased from Frontier Scientific Inc. (Logan, UT, USA). Soybean oil and 2′,7′-dichlorofluorescin diacetate (DCFDA) were purchased from Sigma-Aldrich (St. Louis, MO, USA). Ethyl alcohol was purchased from Duksan (Seongnam, Gyeonggi-do, Korea). Dimethyl sulfoxide (DMSO) and Triton X-100 were purchased from Samchun (Seoul, Gangnam-gu, Korea). Optimal cutting temperature (O.C.T) compound was purchased from Sakura^®^ Finetek (Tokyo, Japan). Hoechst 33342 was purchased from Thermo Fisher Scientific (Waltham, MA, USA). Thiazoyl blue tetrazolium bromide (MTT) was purchased from Biosesang (Seongnam, Gyeonggi-do, Korea). Dulbecco’s phosphate buffered saline (DPBS), Roswell Park Memorial Institute (RPMI) 1640 medium, and fetal bovine serum (FBS) were purchased from Biowest (Nuaille, France). The 0.05% Trypsin-EDTA and antibiotic-antimycotic solution were purchased from Gibco BRL (Grand Island, NY, USA).

### 4.2. Preparation of Nanoemulsion

Ce6-loaded PEG-b-PCL nanoemulsions (Ce6-PCL-NEs) were prepared using the conventional O/W emulsion method. Ce6 (4 mg) and soybean oil (100 mg) were dissolved in 40 μL DMSO. PEG-b-PCL (10 mg) was dissolved in 460 μL ethyl alcohol, and the solution was stirred for 30 min at 60 °C until the color became transparent. Each solution in DMSO and ethyl alcohol were added to 2.5 mL distilled water at 80 °C. The mixed solution was sonicated using a probe sonicator (Sonic & Materials Inc., Newtown, CT, USA) for 5 min. The resulting solution was passed into the chamber of Microfluidics LV1 (Westwood, MA, USA). Unloaded Ce6 and DMSO were removed by dialysis (MWCO: 13KD) in distilled water for one hour.

### 4.3. Characterization of Nanoparticles

The size and zeta potential of the Ce6-PCL-NEs were determined with Zetasizer Nano ZS90 (Malvern Instruments, Malvern, UK) at 25 °C in PBS (pH 7.4). The morphology of the Ce6-PCL-NEs was studied by transmission electron microscopy (TEM) utilizing a negative stain containing 2% (*w/v*) uranyl acetate solution. To monitor the release of Ce6 from Ce6-PCL-NEs, Ce6-PCL-NEs in a dialysis membrane (MWCO: 13KD) were positioned in DPBS (pH 7.4). After we obtained the release medium at a predetermined time point, it was fully dissolved in a detergent solution (DMSO: PBS: DW = 5: 4: 1, 1% Triton X-100). We measured the amount of the released Ce6 (405/650 nm) fluorescence using a synergy H1 Hybrid Multi-Mode Reader (Biotek Instruments, Inc., Winooski, VT, USA). The intensity of released Ce6 was calculated in comparison with the fluorescence of Ce6 (405/650 nm). The Ce6 encapsulation efficiency (EE) of the Ce6-PCL-NEs was calculated as EE (%) = Amount of Ce6 in the Ce6-PCL-NEs/Total amount of Ce6 added × 100%

### 4.4. Cellular Uptake and DCFDA Assay

In vitro studies were performed with the 4T1 mouse breast cancer cells. 4T1 cells were obtained from the American Type Culture Collection (Rockville, MD, USA) and cultured in RPMI medium with 10% fetal bovine serum (FBS) and 1% antibiotic-antimycotic solution. First, 4T1 cells were seeded at 2 × 10^4^ cells per well in 24-well plates and incubated for 2 days. The cells were washed with DPBS and treated with different concentrations of free Ce6 or Ce6-PCL-NEs (2–8 μg/mL) in serum-free medium without FBS and incubated for 2 h. After washing with DPBS, the cells were stained with Hoechst 33342 (2 μg/mL) for 15 min. After washing with DPBS, imaging of cellular uptake was performed with a fluorescence inverted microscope IX 71 (Olympus, Tokyo, Japan). We used 1% DMSO in case of free Ce6 group during in vitro and in vivo experiments.

Reactive oxygen species generation of free Ce6 and Ce6-PCL-NEs was measured by DCFDA. Each sample was added to pre-incubated cells on the 24-well plate. After incubating for 2 h, the cells were washed with DPBS, and then DCFDA and Hoechst 33342 were added for 30 min. Each well was irradiated with a 671-nm laser and imaged by an Axiovert 200 fluorescence inverted microscope (Carl Zeiss, Oberkochen, Germany).

### 4.5. Cell Viability with or without Laser Irradiation

The 4T1 cell viability was measured by MTT (3-(4,5-dimethylthiazol-2-yl)-2,5-diphenyltetrazolium bromide) assay. The cells were seeded into a 96-well plate at 5 × 10^3^ cells per well and incubated for 1 day. After washing with DPBS, the cells were treated with free Ce6 or Ce6-PCL-NEs in serum-free media for 2 h. After again washing with DPBS (pH 7.4), the used media was replaced with fresh. To monitor dark toxicity, all of the wells were treated with MTT solution and incubated for 2 h. After elimination of the remaining solution, DMSO was added to each well, and then the absorbance was measured at 570 nm with a synergy H1 Hybrid Multi-Mode Reader (Biotek Instruments, Inc., Winooski, VT, USA). To investigate the photodynamic therapy effect in vitro, all of the wells were irradiated by a 671-nm laser and treated with MTT solution. Afterward, the following processes were performed as described above for dark toxicity experimental steps.

### 4.6. In Vivo and Ex Vivo Imaging

The animal study was approved by the institutional review board of our university (approval No. CUMS-2016-0315-04). Female Balb/c mice (4 weeks old, OrientBio, Seongnam City, Korea) were used for in vivo and ex vivo imaging. To make a tumor-bearing mouse model (*n* = 3 per each group), 1.5 × 10^6^ 4T1 cells in culture medium (100 μL) were injected subcutaneously into the left flank region. After tumors grew to approximately 150 ± 30 mm^3^, free Ce6 or Ce6-PCL-NEs (2.5 mg/kg of Ce6 in 100 μL physiological saline) were intravenously injected into the tail of the mice. The mice were then anesthetized with isoflurane by a respiratory route. Whole body images of all mice were obtained with IVIS Lumina XRMS (PerkinElmer Inc, Waltham, MA) set at excitation 660 nm and emission 710 nm (Cy5.5 filter) at 1, 3, 6, 9, and 12 h postinjection. To obtain blood samples, the mouse tails were cut at given time points (1, 3, 6, 9 and 12 h), and blood was collected into a 384-HT plate and mixed with detergent solution (DMSO:DW = 4:1, 2% Triton X-100). Then, the fluorescence intensity of each well was measured with an IVIS Lumina XRMS. Twelve hours after injection of the samples, the tumors and major organs (heart, lung, liver, spleen, and kidney) were resected, and images were acquired using IVIS Lumina XRMS.

The resected tumor samples were gathered with O.C.T compound in molds and stored at −80 °C. The tumor was cut into 6-μm-thick slices that were then washed with DPBS and stained with Hoechst 33342 (2 μg/mL) for 10 min. Then, fluorescence imaging was carried out using a fluorescence inverted microscope IX71.

To make a metastasis model, 4T1 cells were injected subcutaneously into the left flank region. When the tumor size exceeded 1000 mm^3^, Ce6-PCL-NEs (2.5 mg/kg) were intravenously injected into the mice via the tail vein. After 12 h, the mice were sacrificed, and the organs and tumors were analyzed.

### 4.7. Statistics

The data were analyzed using two sample t-test. In figures, * and *** indicates *p* < 0.05 and *p* < 0.001, respectively.

## 5. Conclusions

In summary, we developed stable nanoemulsions using soybean oil and PEG-b-PCL as an amphiphilic surfactant. They were fabricated by heating and cooling based on the low melting point of PCL. For PDT, Ce6 was loaded into the oil core as PS. The resulting Ce6-PCL-NE showed a spherical nanostructure of about 220.3-nm diameter and near neutral surface charge of −0.564 mV. It showed fast cellular uptake and ROS generation in 4T1 tumor cells upon laser irradiation. After intravenous injection into 4T1 tumor-bearing mice, it showed increased blood circulation time and higher accumulation in tumor tissue compared to free Ce6. In particular, Ce6-PCL-NE also showed efficient drug delivery to metastatic tumor tissue in lung and other sites. These overall results demonstrate the promising potential of Ce6-PCL-NE as a drug carrier for PDT and in vivo drug delivery.

## Abbreviations

PEG-b-PCL	poly(ethylene glycol)-block-polycaprolactone
PDT	photodynamic therapy
PS	photosensitizer
Ce6	chlorin e6
NE	nanoemulsion
NP	nanoparticle
ROS	reactive oxygen species
EPR	enhanced permeability and retention
